# On the adsorption characteristics and mechanism of methylene blue by ball mill modified biochar

**DOI:** 10.1038/s41598-023-48373-1

**Published:** 2023-12-01

**Authors:** Jinxia Wang, Yunfeng Tan, Hongjun Yang, Lingling Zhan, Guowen Sun, Le Luo

**Affiliations:** 1 College of Resources and Safety, Chongqing Vocational Institute of Engineering, Chongqing, 402260 China; 2 College of River and Ocean Engineering, Chongqing Jiaotong University, Chongqing, 400074 China; 3 College of Resources and Environment, Southwest University, Beibei, Chongqing, 400715 China

**Keywords:** Environmental sciences, Environmental chemistry, Environmental impact

## Abstract

In this study, modified biochar (BRB) was prepared from rice straw by ball milling technique and used for the adsorption of methylene blue (MB) in wastewater. The BRB was characterized by SEM, FTIR and XPS, and the adsorption model and Box–Behnken design were used to optimize the five influencing factors. The results showed that the ball milling technique could increase the content of functional groups (–OH, C=C and C–O, etc.) and aromatic structures on the surface of biochar, thus facilitating the removal of MB. The isotherm model was consistent with the Langmuir adsorption model (R^2^ = 0.947) and the maximum adsorption capacity was 50.27 mg/g. The adsorption kinetics was consistent with the pseudo-second-order kinetic model (R^2^ = 1) and the adsorption rate was mainly controlled by chemisorption. The thermodynamic model confirmed that the adsorption process was a spontaneous heat absorption reaction. The maximum adsorption efficiency was 99.78% under the optimal conditions (40℃, pH 8, reaction time = 90 min, dosing amount = 0.1 mg), and the adsorption efficiency could be improved by increasing the pH and BRB dosing amount. The surface functional groups and crystal structure properties of BRB were the main determinants of adsorption, and it was clarified that physical adsorption, electrostatic attraction and π-π interaction were the main mechanisms for the adsorption of MB by BRB. The main mechanisms were clarified. Therefore, BRB is an economic, efficient and green adsorption material with good potential for the removal of dye pollutants in the aqueous environment.

## Introduction

Dyes are colored aromatic organic compounds that absorb light and give color to the visible area. It is widely used in textile, food, rubber, printing, cosmetics, pharmaceutical, plastic, concrete and paper industries^1,2^. The textile industry is the first application field that generates a significant amount of wastewater from dyeing^3^. If wastewater is discharged directly, the dyes in the wastewater can interfere with the growth of aquatic plants by enhancing the absorption of sunlight, thereby reducing their photosynthetic activity and causing serious ecological problems in the environment^4,5^. The most commonly used MB dye is a cationic heterocyclic compound with a chemical formula of C_16_H_18_N_3_SCl^6,7^. It was first synthesized in 1876 by Badische Anilin and Soda Fabrik's Heinrich Caro as an aniline-based synthetic dye and is widely used in the dyeing process of the textile industry. According to research findings, it has been shown that under certain conditions, MB can decompose and produce harmful gases such as carbon monoxide, carbon dioxide, nitrogen oxides, sulfur oxides, and so on. Long-term exposure to MB can cause methemoglobinemia, diarrhea, allergic dermatitis and cancer^8^. Therefore, people have done a lot of research on the removal of MB in wastewater^6,9,10^. The primary methods for removing dyes from wastewater include photocatalytic degradation, ion exchange, adsorption, coagulation, oxidation, and biological treatment^11^. However, adsorption is considered to be one of the most sustainable methods for dye removal because of its simple design, economic feasibility, high efficiency and its environmental friendliness^12–14^.

Biochar (BC) is a carbon-rich solid product obtained by biomass pyrolysis under anoxic or anaerobic conditions^15^. In recent years, it has been widely used as an adsorbent in wastewater treatment and soil remediation^16,17^. The raw materials of BC mainly come from agricultural and forestry waste, industrial waste and animal manure^18^. The physical and chemical properties of BC are mainly affected by the source of raw materials^19^. Among them, lignin and cellulose extracted from agricultural and forestry waste can form porous structure, which is conducive to the removal of pollutants through pore filling effect^20^. However, the widespread cultivation of rice means that its straw is not only a rich biomass raw material, but also contains high silica, which provides structural advantages in the preparation of BC^21^. Nevertheless, the original BC still has some limitations in adsorbing dye pollutants^22^. In order to overcome this deficiency, the method of surface modification has gradually become a hot topic for researchers in recent years, and a large number of physical and chemical modification technologies have emerged^23^. Among them, ball milling modification is a method of mechanical ball milling of biochar materials, which changes the structure of adsorption materials by reducing the particle size of BC particles, thereby improving the overall performance of biochar^24,25^. Xiang, W et al.^26^ prepared hickory wood biochar to adsorb acetone by ball milling technology. The adsorption efficiency was 13.0 times higher than that before ball milling, and the maximum adsorption capacity of acetone reached 103.4 mg/g. Panahi, The research conducted by Amusat et al.^27^ demonstrates that ball milling modification technique can enhance the specific surface area, porosity, and pore distribution of wheat straw biochar. It can even influence the structural changes of functional groups. However, there are relatively few studies on MB adsorption by ball milling modified biochar, and the adsorption mechanism is not yet clear.

Therefore, in this paper, rice straw was used as raw material to prepare biochar (RB), and rice ball milling biochar (BRB) was obtained by ball milling modification and used for MB adsorption research. The impact of key parameters (initial methylene blue concentration, BRB dosage, and pH) and their interaction on the removal efficiency of methylene blue was analyzed using response surface methodology. The samples were characterized by scanning electron microscopy (SEM), energy dispersive X-ray analysis (EDX), Fourier transform infrared spectroscopy (FTIR), X-ray diffraction (XRD) and X-ray photoelectron spectroscopy (XPS). The adsorption mechanism of BRB was discussed by combining adsorption kinetics, adsorption thermodynamics and adsorption isotherm model.

## Materials and methods

### Biochar preparation and reagents

Rice straw was taken from Chongqing, China, and passed through a 20-mesh sieve after being crushed. It was placed in a tube furnace (OTF-1200X, Shenzhen Kejing Zhida Technology Co., Ltd., China). Before the start of pyrolysis, N_2_ (100 mL/min) was introduced for 30 min to eliminate residual air. Continuous introduction of N_2_ for 2 h at a heating rate of 10 ℃/min was conducted at a temperature of 500 ℃. After cooling to room temperature, RB was obtained by washing three times with ultrapure water and drying in an oven at 80℃ for 12 h. RB was milled with a planetary ball mill (MITR-YXQM-2L, Changsha Miqi Instruments Co., Ltd., China) for 30 min (1500 rpm) and passed through a 100-mesh sieve to obtain BRB, which was sealed in a glass bottle for subsequent research.

The test water was ultrapure water (18.2 MΩ·cm) (Labonova Direct Pro, Think-lab, Germany). The chemicals used in this study were all analytically pure. Methylene blue (C_18_H_18_ClN_3_·3H_2_O) was purchased from Guoyao Group Chemical Reagents Co., Ltd., hydrochloric acid (HCl) was purchased from Chengdu Cologne Chemical Co., Ltd., and sodium hydroxide (NaOH) was purchased from Chongqing Wansheng Chuandong Chemical Co., Ltd. HCl and NaOH were configured with ultrapure water into a 0.5 mol/L solution for solution pH adjustment.

### Characterize

The adsorption capacity of adsorbents was primarily influenced by the microstructure of the material, composition of elements, and composition of functional groups. In order to study the structural changes of BRB before and after adsorption, scanning electron microscope (SEM) and X-ray energy dispersive spectrometer (EDS) (ZEISS Gemini 300, OXFORD Xplore, Germany) were used to analyze the morphological characteristics and element types of the samples. At the same time, the functional groups of the samples were analyzed by Fourier transform infrared spectroscopy (FTIR) (Nicolet iS50, Semirfei, USA). The lattice characteristics of the samples were analyzed by X-ray diffractometer (XRD) (Ultma IV, Rigaku, Japan). Cu-Kα was used as the emission source during the test. The tube voltage and tube current were 40 kV and 30 mA, respectively. The scanning angle was 10°–80°, the step size was 0.02°, and the scanning speed was 5°/min. The valence state of the sample elements was analyzed by X-ray photoelectron spectroscopy (XPS) (K-Alpha, Thermo Scientific, USA).

### Experimental design and parameter optimization

The adsorption capacity of adsorbents was primarily influenced by the microstructure of the material. In this study, we used a Box–Behnken composite design model to investigate the effects of MB initial concentration, activated carbon dosage, adsorption time, reaction temperature, and pH as experimental parameters, with adsorption efficiency as the response variable. We also examined the impact of elemental composition and functional group composition. The parameters were optimized to obtain the maximum response value. A 5-factor 3-level design (as shown in Table 1) was used, and the number of trials was n = 45. The equation formula for predicting the response value Y was shown in Eq. (1). The software Design-Expert 13.0.1.0 (Stat-Ease Inc, 2021, USA) was used to analyze the variance of the obtained model to determine the significance of the model and regression coefficients^28^.

1 $$Y={\beta }_{0}+\underset{i=1}{\sum^{k}}{\beta }_{i}{X}_{i}+\underset{i=1}{\sum^{k}}\underset{j=i+1}{\sum^{k}}{\beta }_{ij}{X}_{i}{X}_{j}+\underset{i=1}{\sum^{k}}{\beta }_{ii}{X}_{i}^{2}+\varepsilon $$

where Y is the dependent variable, ${X}_{i}$ , ${X}_{j}$ are the coded values of independent variables ($i$, $j$ = 1, 2, 3, 4, 5), ${\beta }_{0}$ is a constant; ${\beta }_{i}$ are linear coefficients; ${\beta }_{ii}$, ${\beta }_{ij}$ are interaction coefficients;* k* is the number of input variables (5).

**Table 1:** Factors and levels of response surface analysis.

Variables	Unit	Symbol	Variables level		
			Low −1	Center 0	High + 1
MB initial concentration	mg/L	X_1_	50	100	150
BRB addition amount	G	X_2_	0.05	0.1	0.15
Reaction time	min	X_3_	30	90	150
Reaction temperature	°C	X_4_	20	30	40
pH	–	X_5_	6	8	10

### Adsorption model

Based on the results of the experimental design and parameter optimization section, the following experiments are carried out under the optimal parameter conditions:

Adsorption kinetic model: in order to understand the mechanism of adsorption process, pseudo-first-order kinetics, pseudo-second-order kinetics and intraparticle diffusion model were used to fit the data, and the adsorption kinetic parameters were evaluated. The kinetic model formulas are shown in Eqs. (2), (3) and (4) ^29^.

2 $${\mathrm{Pseudo{\text{-}}first{\text{-}}order: } \,\,  \,\, q}_{t}={q}_{e}\left(1-{e}^{-{k}_{1}t}\right)$$

3 $${\mathrm{Pseudo{\text{-}}second{\text{-}}order: } \,\, \,\,  q}_{t}=\frac{{k}_{2}{q}_{e}^{2}t}{1+{k}_{2}{q}_{e}t}$$

4 $${\mathrm{Intraparticle \,\,  diffusion: } \,\,  \,\, q}_{t}={k}_{P}{t}^{1/2}+C$$

Adsorption isotherm model: in order to study the effect of initial concentration of MB on adsorption behavior, Langmuir and Freundlich models were used for fitting. The adsorption isotherm model was shown in Eqs. (5) and (6) ^29^.

5 $${{\text{Langmuir}}: q}_{e}=\frac{{{q}_{m}K}_{L}C}{1+{K}_{L}C}$$

6 $${{\text{Freundlich}}: q}_{e}={K}_{F}{C}_{e}^{1/n}$$

Adsorption thermodynamic model: in order to study the effect of temperature change on adsorption behavior, the adsorption thermodynamic model was used for fitting. The model equations are shown in Eqs. (7) and (8) ^29^.

7 $${lnK}_{d}=\frac{{\Delta S}^{0}}{R}-\frac{{\Delta H}^{0}}{RT}$$

8 $${\Delta G}^{0}=-RTln{K}_{d}={\Delta H}^{0}-T{\Delta S}^{0}$$

In the equation : ${q}_{t}$ refers to the adsorption capacity (mg/g) at t (min); ${q}_{e}$ is the equilibrium adsorption capacity (mg/g); ${k}_{1}$ is the rate constant of pseudo-first-order kinetic equation (mg/g·min); ${k}_{2}$ is the rate constant of pseudo-second-order kinetic equation (g/mg·min), and ${k}_{P}$ is the rate constant of interparticle diffusion model (mg/g·min^1/2^); $C$ is a constant related to the interface thickness. ${q}_{m}$ is the maximum adsorption capacity (mg/g); ${K}_{L}$ is the Langmuir model adsorption constant (L/mg); ${K}_{F}$ is Freundlich model adsorption constant (mg/g(L/mg)^1/n^); $n$ is a non-uniform factor, which is related to the adsorption strength. $R$ is the ideal gas constant, 8.314 J/mol·k; $T$ is the thermodynamic temperature (K) ; ${K}_{d}$ is the adsorption equilibrium coefficient; $\Delta G$ is the free energy change of adsorption (KJ/mol); $\Delta H$ is the standard adsorption heat (KJ/mol); $\Delta S$ is the standard entropy change of adsorption (KJ/K).

### Testing method

Tests were performed according to 2.3 and 2.4. All experiments were carried out in a 100 mL conical flask to prepare the required concentration of 50 mL MB solution, adjust the pH, and then add a certain dose of BRB, placed in a constant temperature water bath oscillator (150 rpm) for reaction, through 0.45 μm filter membrane, the concentration of MB was determined. All experiments were set up in 3 parallel groups, and the average value was used for data analysis. Finally, the adsorption capacity *q*_*e*_ and removal rate *η* (%) are calculated at equilibrium, and the calculation equations were shown in Eqs. (9) and (10).

9 $${q}_{e}=\frac{\left({C}_{0}-{C}_{e}\right)V}{m}$$

10 $$\eta \left(\mathrm{\%}\right)=\frac{\left({C}_{0}-{C}_{e}\right)}{{C}_{0}}\times 100\mathrm{\%}$$

Where, *q*_*e*_ (mg/L) is the adsorption capacity at equilibrium, *η* (%) is the removal, C_0_ (mg/L) and C_e_ (mg/L) are the initial and equilibrium concentrations of MB, and m (g) and V (L) are the mass of BRB and the volume of MB solution, respectively.

### Study on regeneration

In order to evaluate the reusability of BRB as adsorbent, 0.5 g of MB adsorbed BRB was added to 50 mL of 0.1 mol/L NaOH solution, and then regenerated by continuous magnetic stirring for 45 min at room temperature. The regenerated BRB was washed three times with ultrapure water to effectively remove the desorbed MB. The regenerated BRB was repeated for 5 adsorption cycles to determine its effectiveness and stability.

### Statement stating

For the collection, treatment and all experimental research process of rice straw materials, all comply with the relevant institutions, national and international guidelines and legislation, all procedures were conducted in accordance with the guidelines, and for the rice straw collected in the experiment, we have obtained permission too, hereby declared.

## Results and analysis

### Characterize

**Figure 1:**
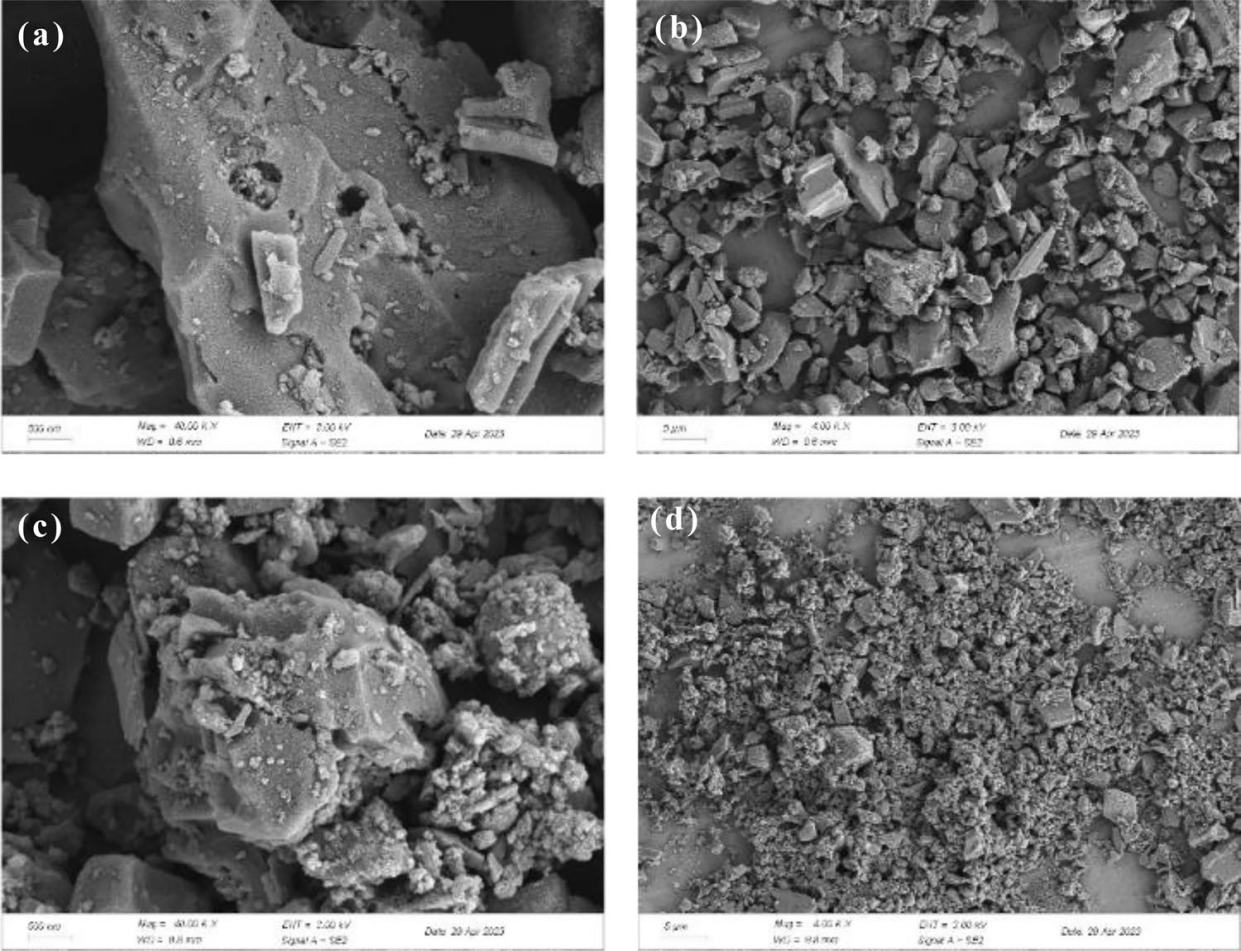
SEM images of PSB [(**a,b**) before adsorption, (**c,d**) after adsorption, at ×40k  and ×4k  magnification).

**Figure 2:**
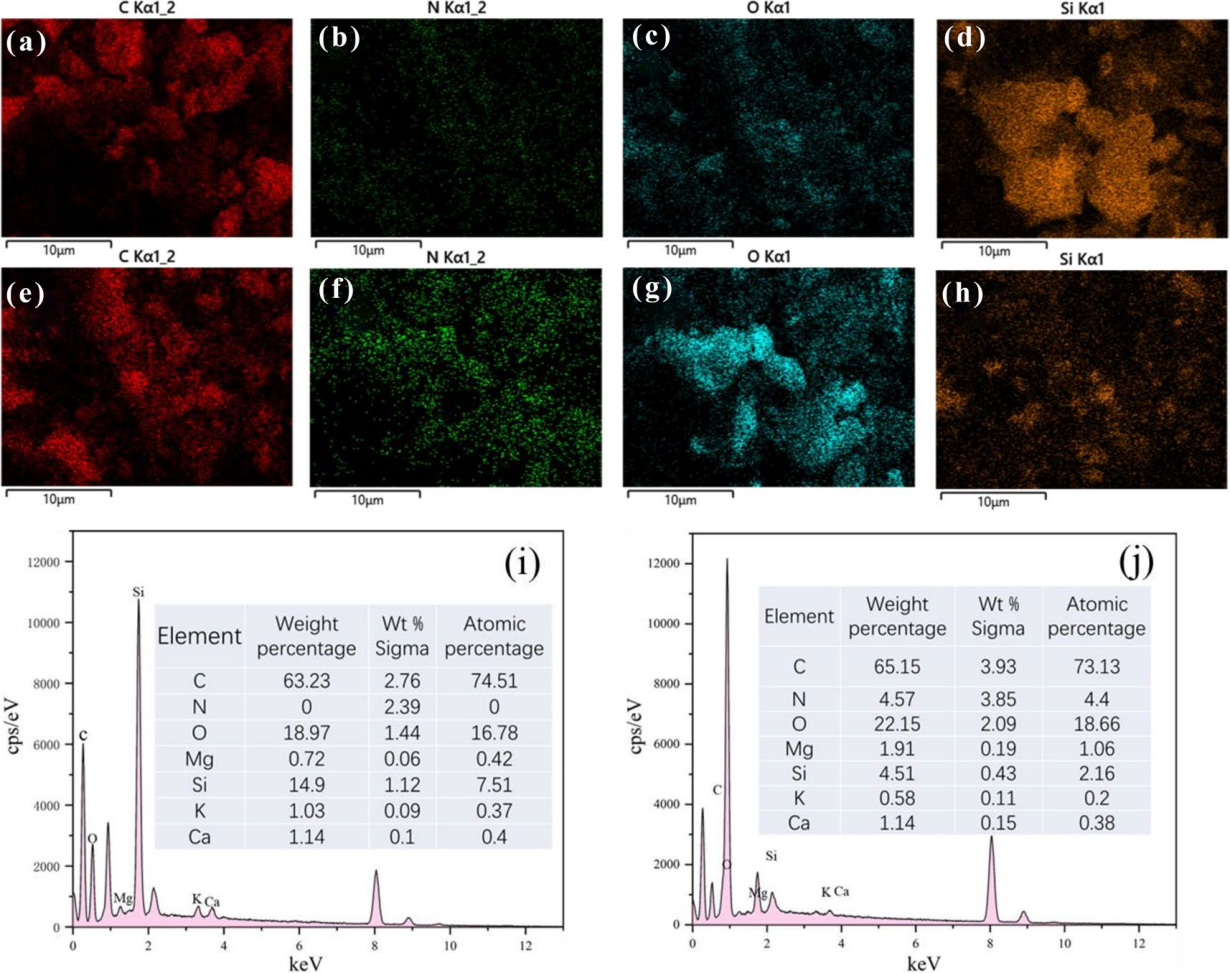
EDX spectra of BRB. (**a–d**), (**i**) BRB before adsorption; (**e–h**), (**j**) BRB after adsorption.

The surface morphology of BRB before and after adsorption was analyzed by SEM. As shown in Fig. 1, the surface morphology of BRB after adsorption was significantly different compared to that before adsorption. Before adsorption BRB had a relatively smooth surface (Fig. 1a, b), while after adsorption BRB had a rough structure (Fig. 1c, d) with a large number of uniformly dispersed microsphere particle aggregates on its surface, indicating that MB was effectively adsorbed by BRB. The EDX spectra before and after adsorption are presented in Fig. 2 and Fig. S1 (see Supporting Materials). EDX elemental analysis confirmed that the main elements of the biochar were C and O, in addition to Si, K, Ca and Mg, all of which changed significantly before and after adsorption, with the most significant change in Si, whose mass fraction decreased from 14.9 to 4.51%, and related studies indicated that these Mineral components were usually present in biochar in the form of carbonates, phosphates and oxides and may influence the sorption potential of significant biochar related^30^. In addition, the presence of elemental N was not detected in the BRB before adsorption, but after adsorption the mass fraction of elemental N in the BRB increased to 4.57%, which further suggests that the adsorption of MB by BRB was significant.

**Figure 3:**
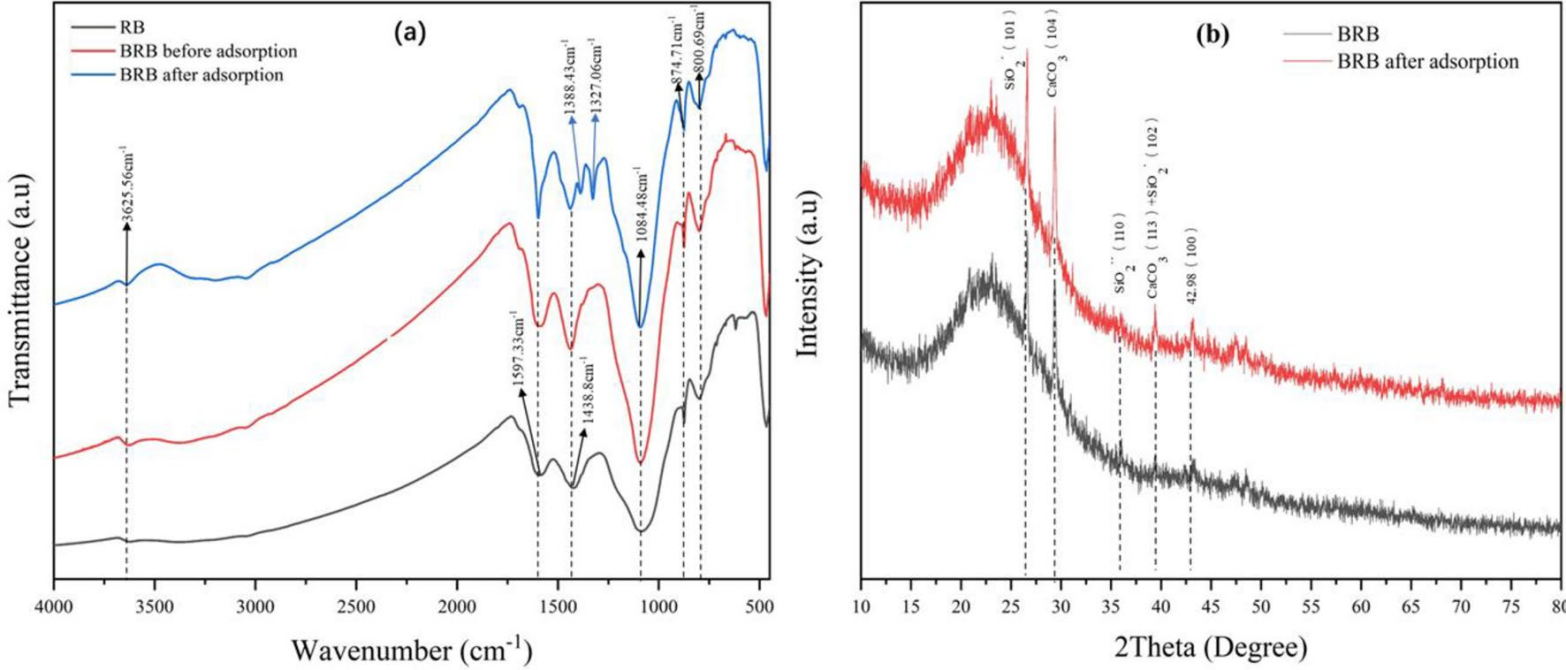
FTIR and XRD of BRB.

The functional groups in RB, BRB and BRB after adsorption were analyzed by FTIR, as shown in Fig. 3a. The vibration intensity of the characteristic peaks in the three materials was quite different, and a new characteristic peak appears in the adsorbed BRB. For RB, the vibration shrinkage of the characteristic peaks of BRB was significantly stronger than that of RB, and after adsorption of MB, the vibration shrinkage of these characteristic peaks becomes significantly weaker. The changes of characteristic peaks at 3625, 1597, 1438, 1084 and 800 cm^−1^ were mainly attributed to the stretching vibration peaks of –OH group, C=C skeleton in aromatic ring, C–H, C–O and aromatic structure^31–35^. However, new characteristic peaks appeared at 1388 and 1327 cm^−1^, which were attributed to the methyl and aromatic nitro groups of MB^36,37^, indicating that MB interacted significantly with BRB functional groups, which was consistent with the Lyu, HH et al.^38^ study. The results show that the material treated by ball milling was more conducive to the removal of MB.

The crystal structure of BRB before and after adsorption was analyzed by XRD. As shown in Fig. 3b, there were characteristic diffraction peaks at 22.62, 26.62, 29.34, 26.04, 29.46, 43.18 and 48.46°. The diffraction peak at 43.18 (100) indicates the presence of aromatic carbon in BRB and the formation of turbine layer crystallites^39^. Additionally, the presence of calcite (CaCO_3_) and quartz (SiO_2_) microcrystalline phases can be observed in the BRB. The formation of CaCO_3_ may be related to the high content of calcium oxalate (CaC_2_O_4_) present in straw^40^. Studies have shown that CaCO_3_ crystals in biochar from many agricultural and forestry wastes (straw, leaves and wood, etc.) were attributed to the formation of calcite during pyrolysis, and the formation of calcite is beneficial to the increase of alkalinity during the preparation of BRB, thus contributing to its removal of MB^41–43^. The formation of calcite was advantageous in increasing the alkalinity during the preparation process of BRB, thereby facilitating the removal of MB.

**Figure 4:**
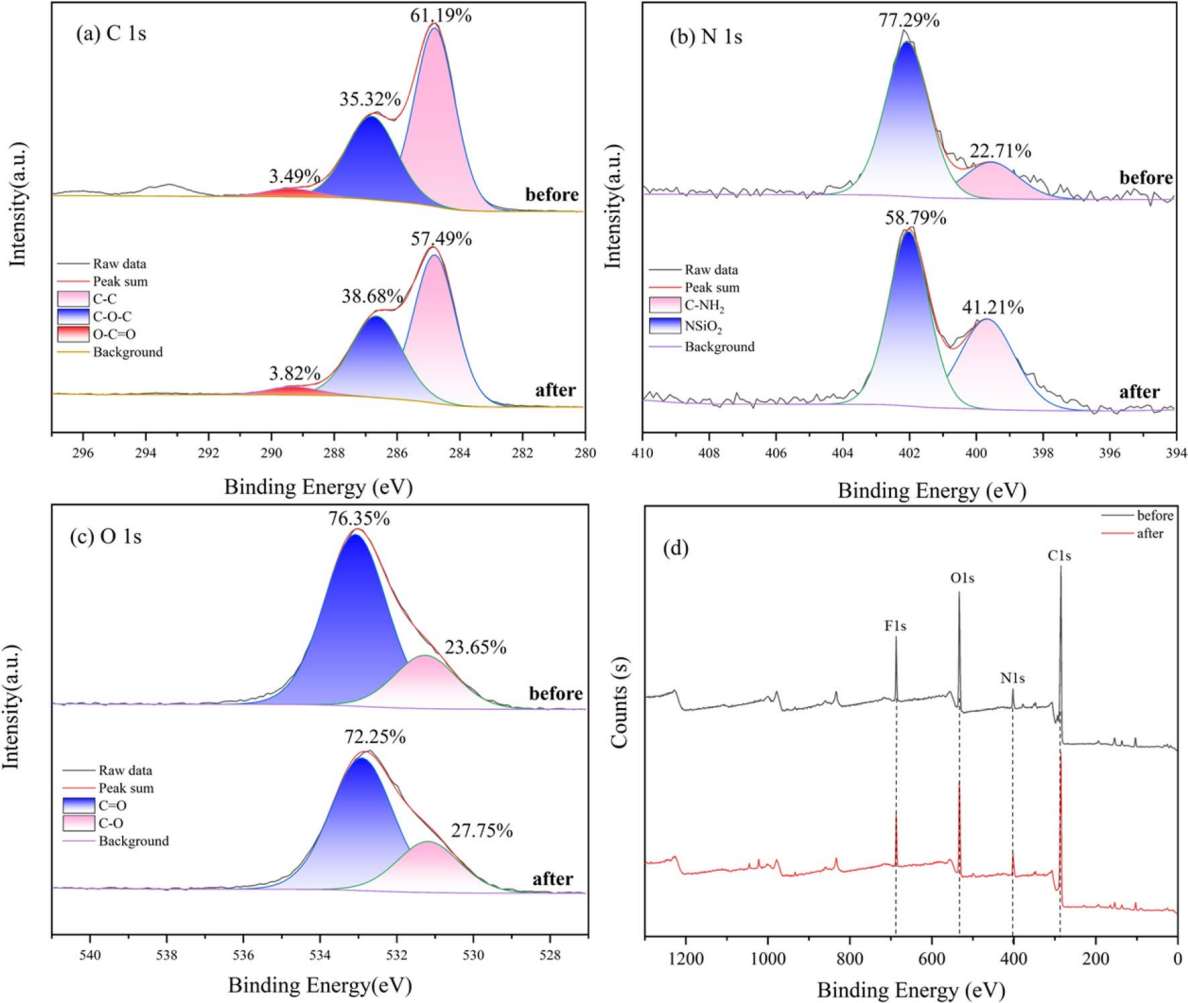
XPS of BRB before and after MB adsorption.

**Figure 5:**
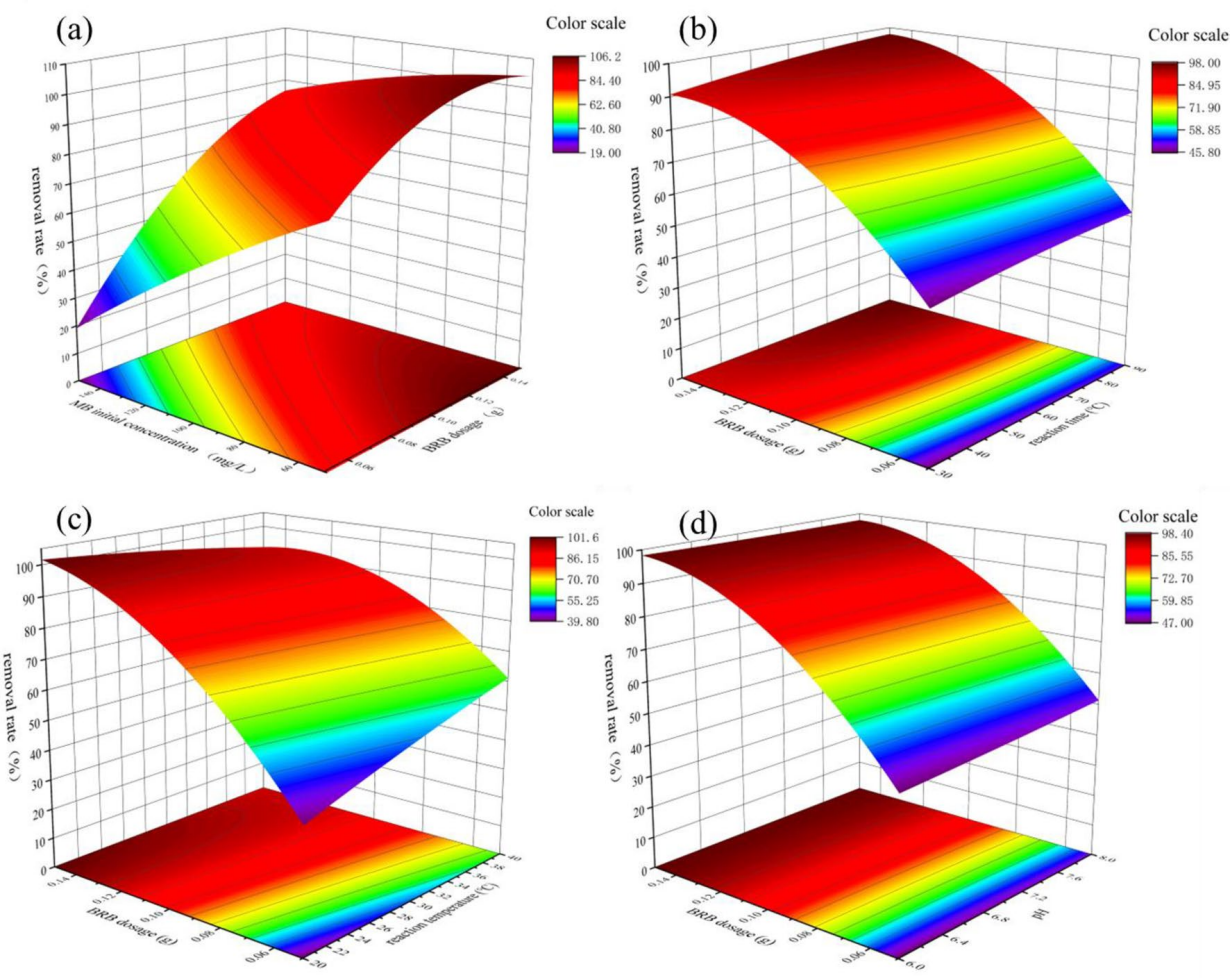
3D response surface (**a**) effect of initial MB concentration and BRB dosage; (**b**) effects of BRB dosage and reaction time; (**c**) the effects of BRB dosage and reaction temperature; (**d**) the 3D diagram of the effects of BRB dosage and pH.

The XPS results before and after MB removal by BRB are shown in Fig. 4. Figure 4a mainly presents three significant peaks caused by carbon (C1 s), nitrogen (N1 s) and oxygen (O1 s). The C1 s spectrum at 284.8, 286.8 and 289.4 eV were divided into three peaks (Fig. 5b), which are attributed to the C–C bond, C–O–C bond and O–C=O bond in aliphatic and aromatic compounds, respectively. After adsorption of MB, the percentage of C–C bond decreased from 66.19% before adsorption to 57.49% after adsorption. In addition, the percentages of C–O–C and O–C=O groups increased from 35.32 to 3.49% to 38.68% and 3.82%, respectively, which was mainly attributed to surface complexation and π–π interaction^44,45^. The binding energies of N1s at 399.7 and 402.1 eV were attributed to N in NSiO_2_ and C–NH_2_ bonds, respectively (Fig. 5c). After adsorption, the percentage of NSiO_2_ bond decreased from 77.29 to 58.79%, which was consistent with the change trend of Si in EDX, indicating that N in MB did not form chemical bonds with Si directly during the adsorption process, but formed n–π interaction with it^46,47^. The percentage of C–NH_2_ bond has increased from 22.71 to 44.21%, which was consistent with the observed change in N based on the EDX analysis results. This can be attributed to the formation of aromatic nitro groups during the adsorption process, which was consistent with the results of FTIR analysis. The successful adsorption of MB has been further confirmed. The binding energies of O1 s at 532.9 and 531.2 eV were attributed to O in C=O bond and C–O bond (Fig. 5d). After the adsorption reaction, the percentage of C=O bond decreased from 76.35 to 72.25%, and the percentage of C–O bond increased from 23.65 to 27.75%. This may be due to the fact that some M–O bonds in the metal oxides of BRB in biochar are converted into M–OH, which leads to an increase in hydroxyl functional groups, and also indicates that oxygen-containing functional groups play an important role in removing MB.

### Experimental design and parameter optimization

The experimental results based on RSM are shown in Table S1. The fitted quadratic polynomial equation was given in Eq. (11):

11 $$ \begin{aligned} {\text{Y}} & = {85}.{21} - {19}.{24} \times {\text{X}}_{{1}} + {23}.{27} \times {\text{X}}_{{2}} + {2}.{34} \times {\text{X}}_{{3}} + {2}.{53} \times {\text{X}}_{{4}} + {2}.{64} \times {\text{X}}_{{5}} + {1}0.0{7} \times {\text{X}}_{{1}} \times {\text{X}}_{{2}} \\ & \quad  + {1}.{83} \times {\text{X}}_{{1}} \times {\text{X}}_{{3}} + 0.{9415} \times {\text{X}}_{{1}} \times {\text{X}}_{{4}} + {2}.{59} \times {\text{X}}_{{1}} \times {\text{X}}_{{5}} + 0.{9379} \times {\text{X}}_{{2}} \times {\text{X}}_{{3}} - {7}.{57} \times {\text{X}}_{{2}} \times {\text{X}}_{{4}}\\ & \quad - {2}.{39} \times {\text{X}}_{{2}} \times {\text{X}}_{{5}} + {1}.{2} \times {\text{X}}_{{3}} \times {\text{X}}_{{4}} + 0.{1617} \times {\text{X}}_{{3}} \times {\text{X}}_{{5}} - 0.{1617} \times {\text{X}}_{{4}} \times {\text{X}}_{{5}} - {2}.{81} \times {\text{X}}_{{1}}^{{2}}\\ & \quad - {11}.{36} \times {\text{X}}_{{2}}^{{2}} - {3}.{88} \times {\text{X}}_{{3}}^{{2}} - 0.{88}0{5} \times {\text{X}}_{{4}}^{{2}} + 0.{7893} \times {\text{X}}_{{5}}^{{2}} \end{aligned} $$

Analysis of variance (ANOVA) (Table S2) was used to determine the statistical significance of all analyses. The statistical significance of the quadratic fit was determined by the LOF, R^2^ and R^2^_adj_ between the predicted and experimental values. The p < 0.0001 indicates that the fitted model is an effective model for predicting experimental values. X_1_, X_2_, X_5_, X_1_ X_2_, X_2_ X_4_ and X_2_^2^ were statistically significant (p < 0.05). Other model terms with p > 0.05 were omitted in the model equation to obtain the best fit for the model. A_deq_ = 24.65 (precision > 4), so there was a good correlation between the experimental and predicted response^48^.

The interaction between variables was studied by 3D response surface. Figure 5a shows that by increasing the amount of BRB from 0.05 to 0.15 g, the removal rate increased from 29.90 to 99.65% with the decrease of MB initial concentration. This can be attributed to an increase in the number of adsorption sites of MB as the dosage increases^49^. Figure 5b shows that by increasing the reaction time from 30 to 90 min, the MB removal rate increases significantly, which may be that MB needs enough time to diffuse more fully inside the BRB^50^. From Fig. 5c, it can be observed that as the temperature increases from 20 to 40 ℃, the removal efficiency of MB increases from 56.18 to 86.71%. This indicates that temperature has a significant impact on the removal of MB. Figure 5d shows that pH has a significant effect on MB removal rate. When the pH value was greater than the zero point of BRB (pH_zpc_), the BRB surface will obtain negative charge and promote the adsorption of MB by electrostatic attraction^51^, as described in the following equation.

12 $${\text{BRB}}-{{\text{O}}}^{-}+{{\text{MB}}}^{+}\leftrightarrow {\text{BRB}}-{{\text{O}}}^{-}{\cdots \cdots }^{+}{\text{MB}}$$

### Model investigation

**Figure 6:**
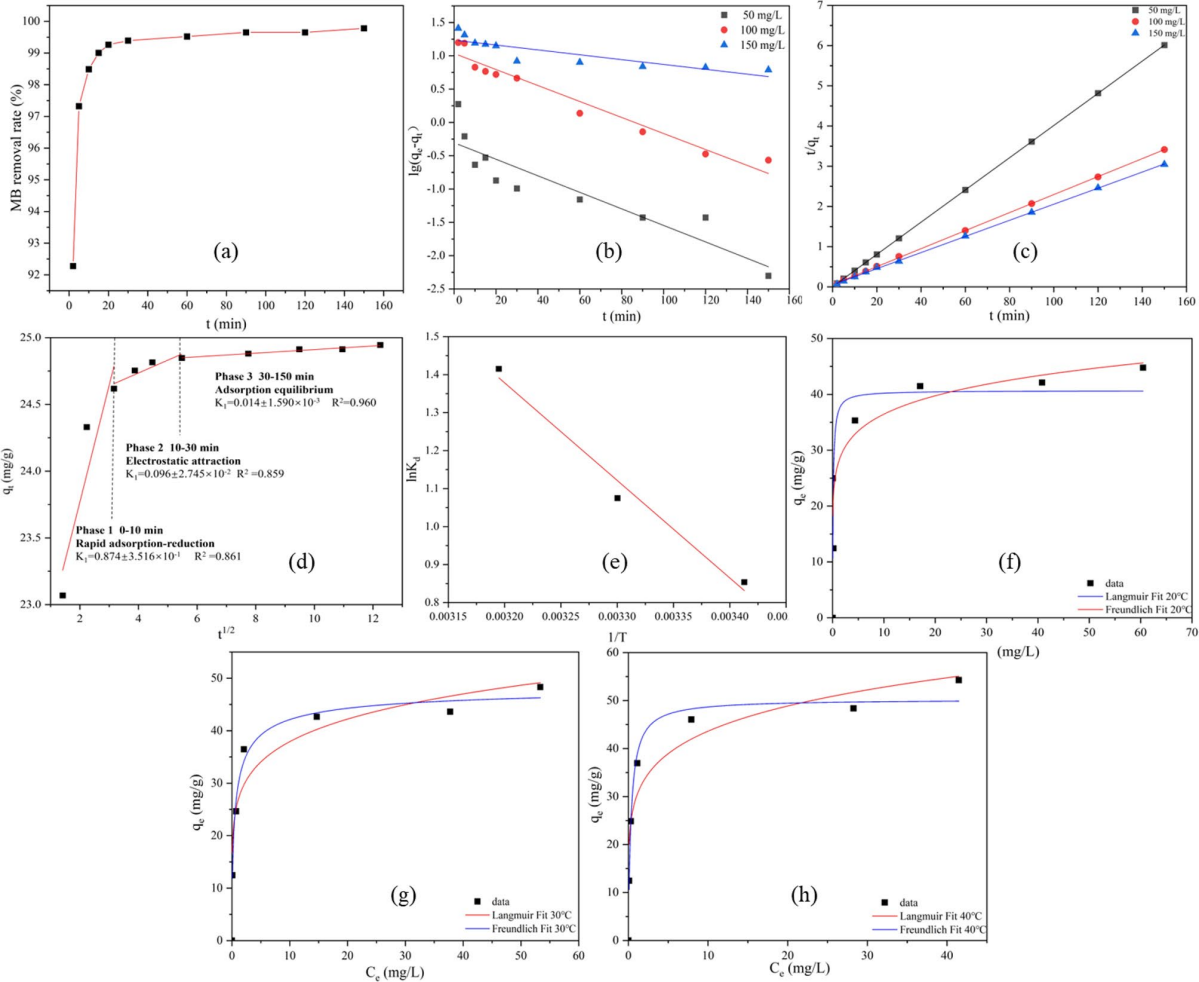
MB adsorption model of BRB: (**a**) t-adsorption rate curve; (**b–d**) pseudo-first-order, pseudo-second-order dynamic model and particle diffusion model; (**e**) thermodynamic model; (**f–h**) isotherm model (BRB dosage = 0.1 g, MB initial concentration = 100 mg/g; temperature = 20, 30 and 40 ℃; pH  7, reaction time = 180 min).

The experimental data were simulated by kinetic models (pseudo-first-order, pseudo-second-order and inter-particle), and the adsorption process of MB on BRB was analyzed. The simulation results for the adsorption of MB on BRB using three different kinetic models are shown in Fig. 6. Figure 6a illustrates the effect of contact time on the removal of MB from BRB, indicating that the initial 20 min constitute a rapid adsorption phase followed by an adsorption equilibrium phase. And when the adsorption equilibrium was reached, it tends to be stable immediately. This rapid adsorption was due to the fact that BRB provides relatively more adsorption sites at the initial stage of adsorption. Using the pseudo-first-order and pseudo-second-order models (Fig. 6a, b). When the initial concentration of MB was 50, 100 and 150 mg/L, R^2^ reaches 1, 0.9998 and 0.9997, respectively, while the pseudo-first-order model was 0.7434, 0.9508 and 0.8285 (Tables S3, S4). The pseudo-second-order model was superior to the pseudo-first-order model. This was because chemical adsorption controls the rate of the adsorption process^52^.

The adsorption behavior was mainly manifested as surface adsorption and pore adsorption. In order to comprehend the adsorption behavior of BRB, a particle internal diffusion model was employed. As shown in Fig. 6d, the adsorption process was divided into three stages. Among them, the value of K was not zero, and the curve does not pass the origin, so the mechanism was not the only limiting process. The adsorption of MB on BRB was a complex multi-factor control process^53–55^. In the first stage of adsorption, the K_1_ value was high (K_1_ = 0.874), indicating that the adsorption of MB mainly occurs on the BRB surface and diffuses faster. In the second stage, as the adsorption sites on the surface of BRB are gradually occupied, MB gradually diffuses into the internal pores of biochar. Since the K_2_ value (K_2_ = 0.096) was significantly lower than that in the first stage, the adsorption efficiency was limited by the intraparticle diffusion, resulting in the internal diffusion efficiency lower than the surface diffusion^56,57^. In the third stage, the K_3_ value decreased to a lower value (K_3_ = 0.014), which was a slow intraparticle diffusion process due to the occupation of intraparticle space.

The temperature has a significant effect on the removal effect. Figure 6e and Fig. S5 were the thermodynamic models and parameters of MB adsorption by BRB. When the (ΔG°) value was less than zero (−2.08, −2.71 and −3.68), the adsorption rate of MB by BRB increases with the increase of temperature, indicating that this was a spontaneous adsorption process^58^. When ΔH° was greater than zero (21.35 kJ/mol), the adsorption process was an endothermic reaction. ΔS° was greater than zero (79.79 kJ/mol), indicating the affinity of BRB to MB. Therefore, the adsorption of MB on BRB was an endothermic reaction of spontaneous adsorption.

In addition, the Langmuir and Freundlich isothermal models (Fig. 6f–h) were analyzed. The fitting parameters are shown in Fig. S6.

As the temperature increases, the Langmuir model (R_2_ = 0.974) outperforms the Freundlich model (R_2_ = 0.898), indicating a greater tendency for dye adsorption to occur through a monolayer process^59^. The adsorption capacity increased with the increase of temperature, and the maximum adsorption capacity of Langmuir was 50.270 mg/g at 40 ℃.

### Regeneration research

The regeneration capacity of adsorbents was considered to be a major parameter for economic and environmental applications. The results of the adsorption–desorption cycle of BRB shown in Fig. S2 show that the regenerated adsorbent can be reused for at least four adsorption–desorption cycles without changing its effectiveness. However, the observed decrease in adsorption efficiency after the third cycle can be attributed to the deposition of MB molecules on the surface of the BRB during the regeneration process.

### Adsorption mechanism

**Figure 7:**
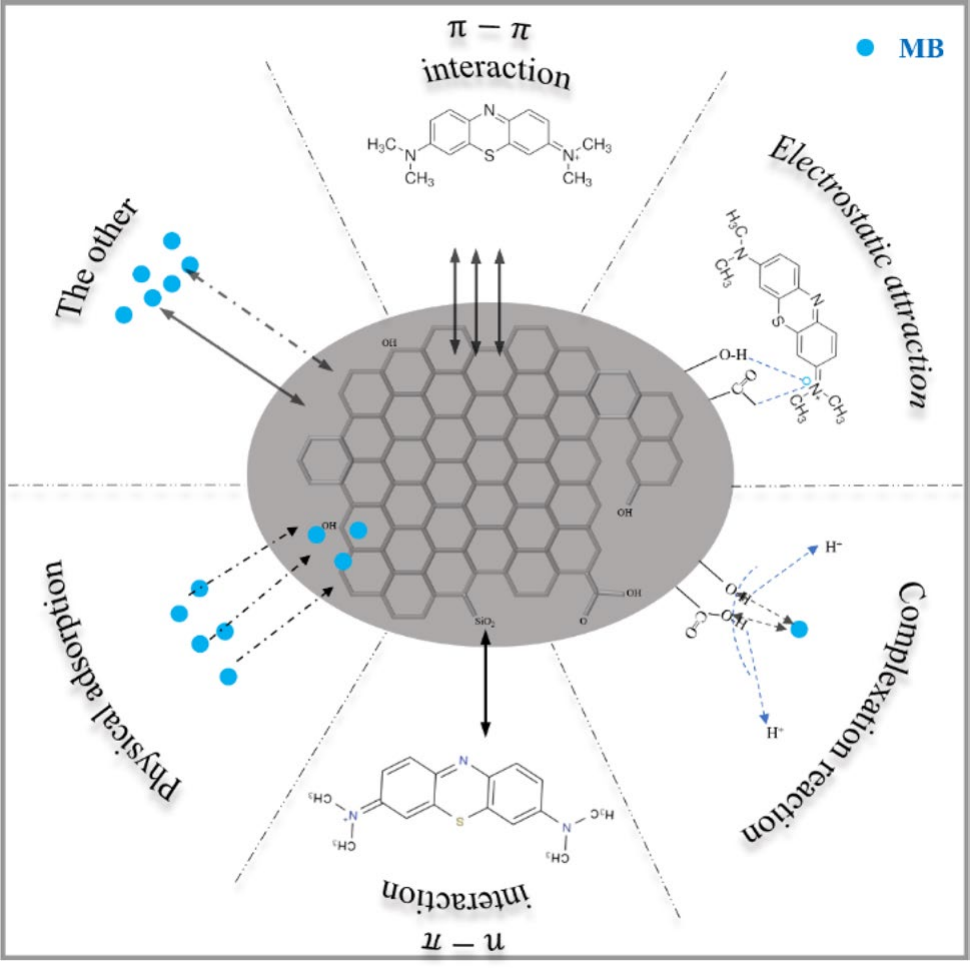
Mechanism diagram of BRB adsorbing MB.

Figure 7 was the adsorption mechanism diagram of BRB on MB, which mainly includes the following mechanisms: (1) Physical adsorption.

The analysis of the SEM image in Fig. 1 reveals the presence of deposits on the surface of the biochar. This suggests that the Binding Reagent BRB effectively removes the MB by undergoing surface adsorption and pore filling, leading to the formation of these deposits^22^. Based on the results of the intraparticle diffusion model, it was shown that among them physical adsorption was the main mechanism for MB removal; (2) the infrared results confirmed the existence of –OH and C=O functional groups in BRB, which could promote the complexation of positively charged MB dye molecules with the surface active groups of BRB^60,61^; (3) when pH > pH_zpc_, the surface functional groups (–OH, –COOH and –NH_3_, etc.) of BRB are deprotonated, and BRB has a good electrostatic attraction to MB. When pH < pH_zpc_, the surface of BRB was protonated, and the adsorption effect of MB cations was significantly reduced due to electrostatic repulsion^62,63^. The response surface analysis results indicate that BRB exhibits the highest adsorption performance at pH 8; (4) The π–π interaction occurs between the π–electron system of the BRB structure and the aromatic group of the MB dye molecule^64^; (5) the presence of Si–O–Si groups was confirmed by n–π interaction, EDX and XPS results, and the presence of Si–O–Si in alkaline solution was beneficial to the adsorption of MB, thus revealing the n–π interaction between the aromatic structure of MB and the Si–O–Si of BRB^65,66^; (6) in other reactions, the hydrogen bonds formed between the free H^+^ on the BRB surface and the O and N in MB are predominantly non-electrostatic, potentially serving as an adsorption mechanism in the reaction system^67,68^. On the other hand, EDX results showed that the contents of various inorganic minerals (Ca^2+^, Mg^2+^ and K^+^, etc.) changed significantly before and after the adsorption of BRB, indicating that these minerals were released from the surface of biochar during the adsorption of MB, allowing ion exchange to occur^69^.

## Conclusions

BRB was prepared using a ball milling technique and the adsorption behavior and mechanism of BRB on MB was analyzed. The successful adsorption of MB on the surface of BRB was confirmed by various characterization techniques. The adsorption kinetic study showed that the adsorption behavior of MB followed pseudo-second order kinetics (R^2^ = 1) and Langmuir isotherm adsorption model (R^2^ = 0.947), and chemisorption was the decisive step for adsorption efficiency with the maximum adsorption capacity of 50.27 mg/g. Thermodynamic analysis showed that the adsorption process of BRB on MB was a spontaneous heat absorption process. The maximum adsorption efficiency reached 99.78% under the optimal conditions (temperature = 40℃, pH 8, reaction time = 90 min, and dosing amount of 0.1 mg). In summary, physical adsorption, electrostatic attraction and π–π interaction may be the main mechanisms for the adsorption of MB by BRB. Therefore, it provides a simple and cost-effective way to achieve recycling of agricultural waste and water purification of organic pollutants.

### Supplementary Information

Supplementary Information.

## Data Availability

The data used to support the findings of this study are available from the corresponding author upon request.
