# Supplementary material for: On the adsorption characteristics and mechanism of methylene blue by ball mill modified biochar
Source: Sci Rep. 2023 Dec 1;13:21174. doi: 10.1038/s41598-023-48373-1 (PMC10692330; doi:10.1038/s41598-023-48373-1)
Supplement: Supplementary file 1 — Supplementary Information. [file 41598_2023_48373_MOESM1_ESM.docx]

**Supplementary information**

**On the adsorption characteristics and mechanism of methylene blue by ball mill modified biochar**

Jinxia Wang ^1, Co, *^, Yunfeng Tan ^2, Co^, Hongjun Yang ^3, *^, Lingling Zhan ^1^, Guowen Sun ^1^, Le Luo ^1^

^1^Chongqing Vocational Institute of Engineering, Chongqing 402260, China.

^2^College of River and Ocean Engineering, Chongqing Jiaotong University, Chongqing 400074, China.

^3^College of Resources and Environment, Southwest University, Chongqing, 400715, China.

^Co^These authors contributed equally to this work and should be regarded as Co-authors.

* Corresponding author: Jinxia Wang, e-mail: [jinxiawang@cqvie.edu.cn](mailto:jinxiawang@cqvie.edu.cn); Hongjun Yang, e-mail: [meilirensheng@swu.edu.cn](mailto:meilirensheng@swu.edu.cn).

**Content**

**Table S1.** Response surface test results of BRB.

**Table S2.** ANOVA of response surface model for the surface area of BRB.

**Table S3.** MB Pseudo-first-order adsorption kinetic fitting parameters of BRB at different initial concentrations.

**Table S4.** MB Pseudo-second-order adsorption kinetic fitting parameters of BRB at different initial concentrations.

**Table S5.** MB BRB thermodynamic fitting parameters at different temperatures.

**Table S6.** MB BRB isotherm model fitting parameters at different temperatures.

**Table S7.** The best variables and responses of removing MB from BRB.

**Fig. S1.** EDX spectra of BRB. (a) –(d) BRB before adsorption; (e)-(h) BRB after adsorption.

**Fig. S2.** Study on repeatability of MB adsorption by BRB.

**Fig. S3.** Adsorption capacity of RB and BRB for MB.

**Table S1.** Response surface test results of BRB.

| **Std** | **Run** | **Variables and their coded values** | | | | | **MB removal (%)** | |
| --- | --- | --- | --- | --- | --- | --- | --- | --- |
|  |  | **MB initial concentration** | **BRB dosage** | **reaction time** | **reaction temperature** | **pH** | **Yexp** | **adsorption quantity（mg/g）** |
| 12 | 1 | 100 | 0.15 | 90 | 30 | 10 | 99.26 | 33.09 |
| 37 | 2 | 100 | 0.05 | 90 | 20 | 8 | 38.20 | 38.20 |
| 36 | 3 | 150 | 0.1 | 90 | 30 | 10 | 70.72 | 53.04 |
| 34 | 4 | 150 | 0.1 | 90 | 30 | 6 | 60.37 | 45.28 |
| 21 | 5 | 100 | 0.05 | 30 | 30 | 8 | 45.83 | 45.83 |
| 2 | 6 | 150 | 0.05 | 90 | 30 | 8 | 29.90 | 44.85 |
| 24 | 7 | 100 | 0.15 | 150 | 30 | 8 | 99.00 | 33.00 |
| 40 | 8 | 100 | 0.15 | 90 | 40 | 8 | 86.71 | 28.90 |
| 20 | 9 | 100 | 0.1 | 90 | 40 | 10 | 92.66 | 46.33 |
| 22 | 10 | 100 | 0.15 | 30 | 30 | 8 | 96.55 | 32.18 |
| 32 | 11 | 100 | 0.1 | 150 | 30 | 10 | 90.08 | 45.04 |
| 6 | 12 | 100 | 0.1 | 150 | 20 | 8 | 78.82 | 39.41 |
| 7 | 13 | 100 | 0.1 | 30 | 40 | 8 | 81.41 | 40.71 |
| 15 | 14 | 50 | 0.1 | 150 | 30 | 8 | 99.52 | 24.88 |
| 39 | 15 | 100 | 0.05 | 90 | 40 | 8 | 56.18 | 56.18 |
| 41 | 16 | 100 | 0.1 | 90 | 30 | 8 | 86.71 | 43.36 |
| 5 | 17 | 100 | 0.1 | 30 | 20 | 8 | 74.94 | 37.47 |
| 8 | 18 | 100 | 0.1 | 150 | 40 | 8 | 90.08 | 45.04 |
| 28 | 19 | 150 | 0.1 | 90 | 40 | 8 | 64.40 | 48.30 |
| 33 | 20 | 50 | 0.1 | 90 | 30 | 6 | 99.65 | 24.91 |
| 44 | 21 | 100 | 0.1 | 90 | 30 | 8 | 82.19 | 41.09 |
| 27 | 22 | 50 | 0.1 | 90 | 40 | 8 | 99.78 | 24.95 |
| 38 | 23 | 100 | 0.15 | 90 | 20 | 8 | 99.00 | 33.00 |
| 19 | 24 | 100 | 0.1 | 90 | 20 | 10 | 86.58 | 43.29 |
| 4 | 25 | 150 | 0.15 | 90 | 30 | 8 | 84.66 | 42.33 |
| 23 | 26 | 100 | 0.05 | 150 | 30 | 8 | 44.54 | 44.54 |
| 25 | 27 | 50 | 0.1 | 90 | 20 | 8 | 99.52 | 24.88 |
| 16 | 28 | 150 | 0.1 | 150 | 30 | 8 | 55.77 | 41.83 |
| 10 | 29 | 100 | 0.15 | 90 | 30 | 6 | 98.62 | 32.87 |
| 43 | 30 | 100 | 0.1 | 90 | 30 | 8 | 83.09 | 41.55 |
| 31 | 31 | 100 | 0.1 | 30 | 30 | 10 | 81.54 | 40.77 |
| 30 | 32 | 100 | 0.1 | 150 | 30 | 6 | 83.09 | 41.55 |
| 45 | 33 | 100 | 0.1 | 90 | 30 | 8 | 86.71 | 43.36 |
| 14 | 34 | 150 | 0.1 | 30 | 30 | 8 | 48.44 | 36.33 |
| 17 | 35 | 100 | 0.1 | 90 | 20 | 6 | 82.45 | 41.22 |
| 18 | 36 | 100 | 0.1 | 90 | 40 | 6 | 89.17 | 44.59 |
| 3 | 37 | 50 | 0.15 | 90 | 30 | 8 | 99.65 | 16.61 |
| 13 | 38 | 50 | 0.1 | 30 | 30 | 8 | 99.52 | 24.88 |
| 35 | 39 | 50 | 0.1 | 90 | 30 | 10 | 99.65 | 24.91 |
| 1 | 40 | 50 | 0.05 | 90 | 30 | 8 | 85.16 | 42.58 |
| 9 | 41 | 100 | 0.05 | 90 | 30 | 6 | 40.53 | 40.53 |
| 42 | 42 | 100 | 0.1 | 90 | 30 | 8 | 87.36 | 43.68 |
| 11 | 43 | 100 | 0.05 | 90 | 30 | 10 | 50.75 | 50.75 |
| 29 | 44 | 100 | 0.1 | 30 | 30 | 6 | 75.20 | 37.60 |
| 26 | 45 | 150 | 0.1 | 90 | 20 | 8 | 60.37 | 45.28 |

**Table S2.** ANOVA of response surface model for the surface area of BRB.

| **Source** | **Sum of squares** | $\boldsymbol{df}$ | **Mean square** | ***F* value** | **P value**  **Prob＞*F*** |  |
| --- | --- | --- | --- | --- | --- | --- |
| Model | 16884.74 | 20 | 844.24 | 33.11 | < 0.0001 | significant |
| X1_ initial concentration (mg·L-1) | 5922.38 | 1 | 5922.38 | 232.26 | < 0.0001 |  |
| X2_ biochar dosage (g·L-1) | 8665.69 | 1 | 8665.69 | 339.84 | < 0.0001 |  |
| X3_ adsorption time (min) | 87.76 | 1 | 87.76 | 3.44 | 0.0759 |  |
| X4_ ℃ | 102.55 | 1 | 102.55 | 4.02 | 0.0563 |  |
| X5_ pH | 111.16 | 1 | 111.16 | 4.36 | 0.0476 |  |
| X1X2 | 405.54 | 1 | 405.54 | 15.9 | 0.0005 |  |
| X1X3 | 13.43 | 1 | 13.43 | 0.5269 | 0.4749 |  |
| X1X4 | 3.55 | 1 | 3.55 | 0.1391 | 0.7125 |  |
| X1X5 | 26.78 | 1 | 26.78 | 1.05 | 0.3157 |  |
| X2X3 | 3.52 | 1 | 3.52 | 0.138 | 0.7135 |  |
| X2X4 | 229.09 | 1 | 229.09 | 8.98 | 0.0062 |  |
| X2X5 | 22.91 | 1 | 22.91 | 0.8985 | 0.3526 |  |
| X3X4 | 5.73 | 1 | 5.73 | 0.2246 | 0.6398 |  |
| X3X5 | 0.1046 | 1 | 0.1046 | 0.0041 | 0.9495 |  |
| X4X5 | 0.1046 | 1 | 0.1046 | 0.0041 | 0.9495 |  |
| X_1_^2^ | 64.43 | 1 | 64.43 | 2.53 | 0.125 |  |
| X_2_^2^ | 1049.52 | 1 | 1049.52 | 41.16 | < 0.0001 |  |
| X_3_^2^ | 122.24 | 1 | 122.24 | 4.79 | 0.0385 |  |
| X_4_^2^ | 6.31 | 1 | 6.31 | 0.2474 | 0.6235 |  |
| X_5_^2^ | 5.07 | 1 | 5.07 | 0.1987 | 0.6597 |  |
| Residual | 611.98 | 24 | 25.5 |  |  |  |
| Lack of fit | 589.19 | 20 | 29.46 | 5.17 | 0.061 | not significant |
| Pure error | 22.78 | 4 | 5.7 |  |  |  |
| Cor total | 17496.72 | 44 |  |  |  |  |
| R²=0.9650，R² adj=0.9359，Adeq Precision=24.65，C.V. % =6.41 | | | | | | |

**Table S3.** MB Pseudo-first-order adsorption kinetic fitting parameters of BRB at different initial concentrations

(The error given by standard deviation).

|  | **Pseudo-first-order dynamic model** | | | **standard error of intercept** | **standard error of slope** |
| --- | --- | --- | --- | --- | --- |
| **MB initial concentration** | **q_e_ (mg/g)** | **K_1_** **(mg/g·min）** | **R^2^** |  |  |
| 50 | 3.43 | 0.00365 | 0.7434 | 0.0537 | 0.000757 |
| 100 | 2.82 | 0.01201 | 0.9508 | 0.06848 | 0.000965 |
| 150 | 0.74 | 0.01241 | 0.8285 | 0.14152 | 0.00200 |

**Table S4.** MB Pseudo-second-order adsorption kinetic fitting parameters of BRB at different initial concentrations

(The error given by standard deviation).

|  | **Pseudo-second-order dynamic model** | | | **standard error of intercept** | **standard error of slope** |
| --- | --- | --- | --- | --- | --- |
| **MB initial concentration** | **q_e_ (mg/g)** | **K_2_ (mg/g·min）** | **R^2^** |  |  |
| 50 | 24.96 | 0.2767 | 1.000 | 0.00084 | 0.00001 |
| 100 | 44.64 | 0.0092 | 0.9998 | 0.00737 | 0.00010 |
| 150 | 49.95 | 0.0076 | 09997 | 0.00801 | 0.00011 |

**Table S5.** BRB thermodynamic fitting parameters at different temperatures.

| **Temp(K)** | **K_d_** | **△G°**  **(KJ/mol)** | **△H°**  **(KJ/mol))** | **△S°**  **(KJ/mol)** | **R^2^** |
| --- | --- | --- | --- | --- | --- |
| 293 | 2.35 | -2.08 | 21.35 | 79.79 | 0.9604 |
| 303 | 2.93 | -2.71 |  |  |  |
| 313 | 4.12 | -3.68 |  |  |  |

**Table S6.** BRB isotherm model fitting parameters at different temperatures.

|  | **Langmuir** | | |  | **Freundlich** | | |
| --- | --- | --- | --- | --- | --- | --- | --- |
| **temperature** | **q_m_ (****mg/g)** | **K_L_ (L/mg)** | **R^2^** |  | **K_F_ (mg/g(L/mg)^1/n^）** | **n** | **R^2^** |
| 20℃ | 40.665 | 8.687 | 0.533 |  | 27.350 | 8.016 | 0.816 |
| 30℃ | 45.320 | 2.098 | 0.946 |  | 26.521 | 6.444 | 0.898 |
| 40℃ | 50.270 | 3.033 | 0.974 |  | 29.904 | 6.100 | 0.898 |

**Table S7**. The best variables and responses of removing MB from BRB.

| **MB initial concentration** | **BRB addition amount** | **reaction time** | **reaction temperature** | **pH** | **removal rate(exp)**  **%** | **removal rate(pre.)**  **%** |
| --- | --- | --- | --- | --- | --- | --- |
| 50 | 0.1 | 90 | 40 | 8 | 99.31 | 100 |


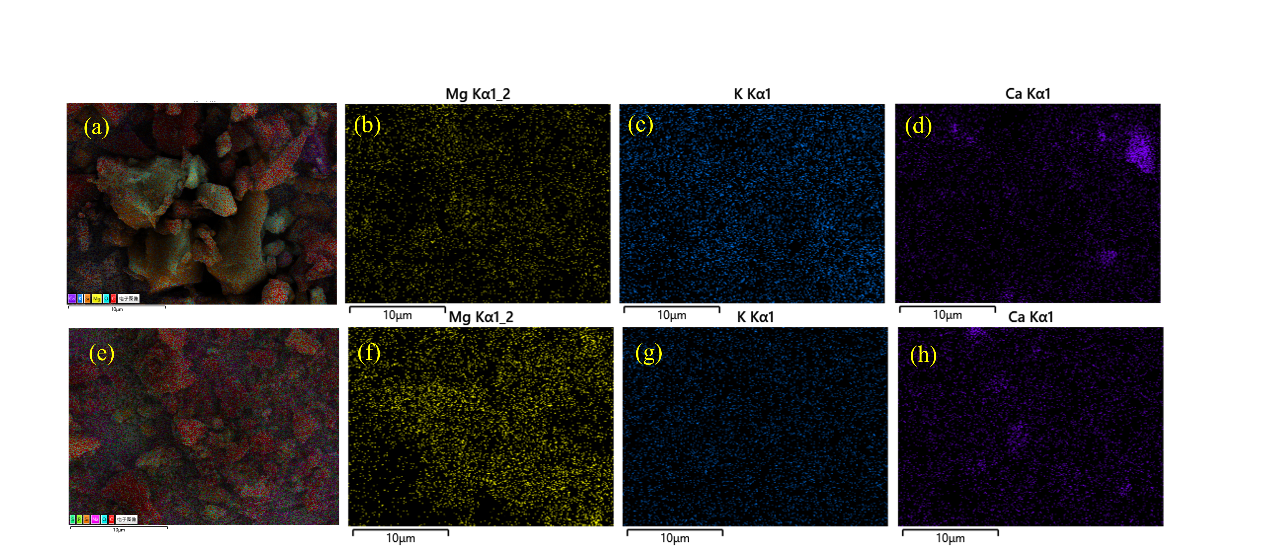


**Figure S1.** EDX spectra of BRB. (a) –(d) BRB before adsorption; (e)-(h) BRB after adsorption.


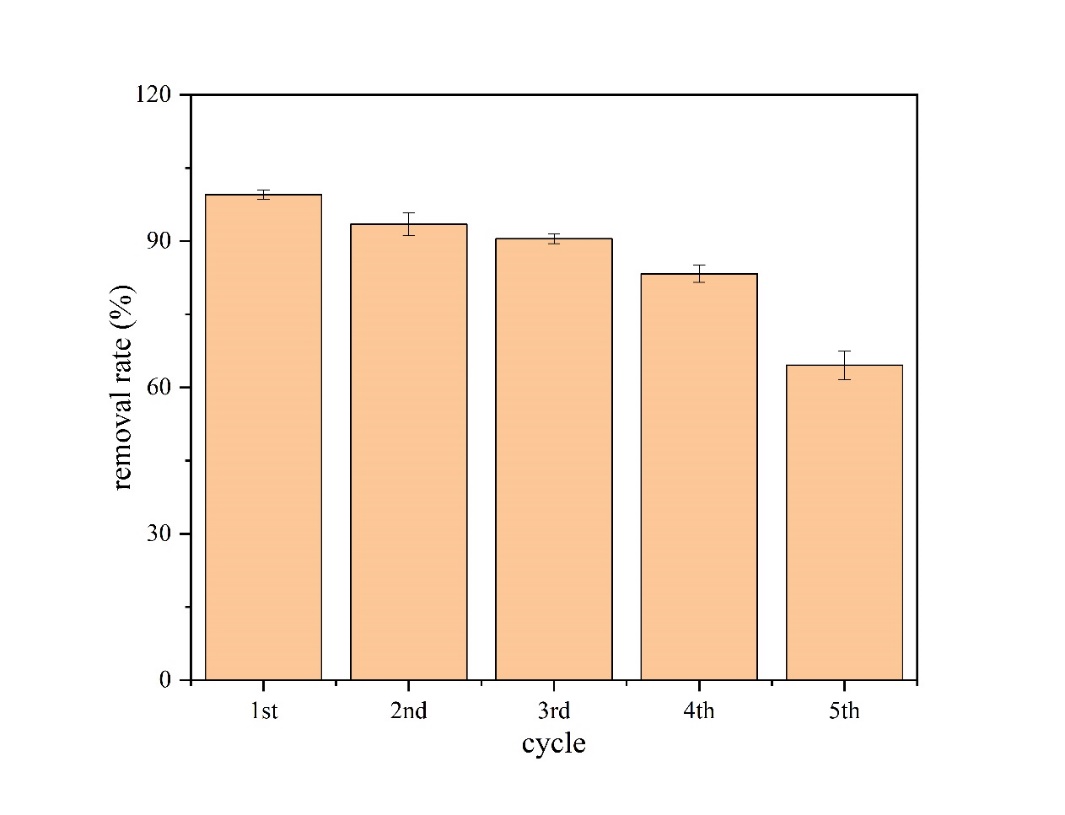


**Figure S2.** Study on repeatability of MB adsorption by BRB.


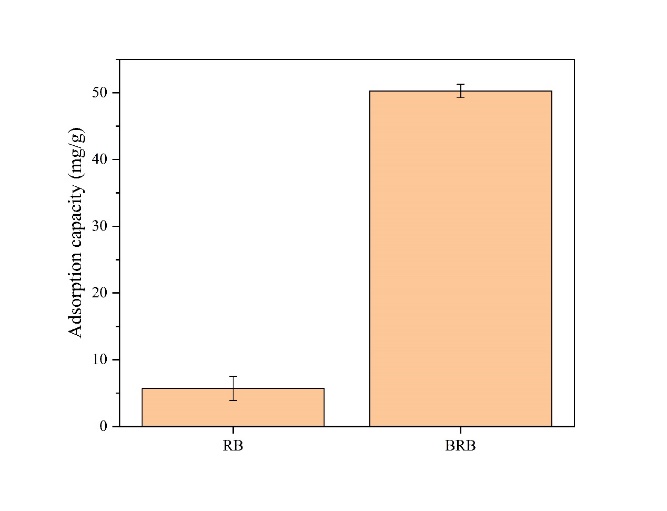


**Figure S3.** Adsorption capacity of RB and BRB for MB
